# Epidemiological and virological characteristics of respiratory tract infections in children during COVID-19 outbreak

**DOI:** 10.1186/s12887-021-02654-8

**Published:** 2021-04-22

**Authors:** Yueling Zhu, Wei Li, Binbin Yang, Ruiying Qian, Fang Wu, Xue He, Qinheng Zhu, Jinling Liu, Yan Ni, Jianbing Wang, Shanshan Mao

**Affiliations:** 1 Department of Traditional Chinese Medicine, The Children’s Hospital, Zhejiang University School of Medicine, National Clinical Research Center for Child Health, National Children’s Regional Medical Center, 310052 Hangzhou, Zhejiang Province PR China; 2 Department of Clinical Laboratory, The Children’s Hospital, Zhejiang University School of Medicine, National Clinical Research Center for Child Health, National Children’s Regional Medical Center, 310052 Hangzhou, Zhejiang Province PR China; 3 Department of General Surgery, The Children’s Hospital, Zhejiang University School of Medicine, National Clinical Research Center for Child Health, National Children’s Regional Medical Center, 310052 Hangzhou, Zhejiang Province PR China; 4 Department of Neurology, The Children’s Hospital, Zhejiang University School of Medicine, National Clinical Research Center for Child Health, National Children’s Regional Medical Center, 3333 Binsheng road, Zhejiang Province 310052 Hangzhou, PR China; 5 Department of Nephrology, The Children’s Hospital, Zhejiang University School of Medicine, National Clinical Research Center for Child Health, National Children’s Regional Medical Center, 310052 Hangzhou, Zhejiang Province PR China; 6 Department of Public Health, Zhejiang University School of Medicine, 310058 Hangzhou, Zhejiang Province PR China; 7 Department of Pulmonology, The Children’s Hospital, Zhejiang University School of Medicine, National Clinical Research Center for Child Health, National Children’s Regional Medical Center, 310052 Hangzhou, Zhejiang Province PR China; 8 Department of Epidemiology and Biostatistics, The Children’s Hospital, National Clinical Research Center for Child Health, Zhejiang University School of Medicine, , 310058 Hangzhou, Zhejiang Province PR China

**Keywords:** COVID-19, Children, Outpatient visits, Respiratory tract infection, Epidemiological characteristics

## Abstract

**Background:**

To investigate the impact of protective measures and isolation on respiratory tract infections in children during the COVID-19 outbreak.

**Methods:**

We extracted data on outpatient visits and respiratory infection visits, and tests of respiratory viruses (adenovirus (ADV), influenza A (FluA), influenza B (FluB) and respiratory syncytial virus (RSV)) from electronic healthcare records in Children’s Hospital, Zhejiang University School of Medicine during the COVID-19 outbreak (January–April, 2020), compared with those in 2018 and 2019 during the same periods.

**Results:**

We found that outpatient visits in January, 2020 was comparable with those in 2018 and 2019, but decreased by 59.9% (288,003 vs. 717,983) and 57.4% (288,003 vs. 676,704), respectively during the period of February-April, 2020, as compared with the same periods in 2018 and 2019. The total number of respiratory tract infections from January to April 2020 decreased by 65.7% (119,532 vs.348,762) and 59.0% (119,532 vs.291,557), respectively compared with the same periods in 2018 and 2019. The proportion of respiratory tract infections during the outbreak also dropped compared with the same periods in 2018 and 2019 (*P*<0.001). We also found significantly decreased number of completed tests for respiratory viruses and positive cases of ADV, FluA, FluB, and RSV during February-April, 2020.

**Conclusions:**

In this study, we found that outpatient visits and respiratory tract infections in children significantly decreased during COVID-19 outbreak. Adequate protective measures and isolation in children may help to prevent respiratory virus infections in children.

## Background

Children are commonly affected by respiratory tract infection which is one of the important causes of pediatric death [1]. It has been confirmed that more than 80 % of acute respiratory infections are caused by viruses. Adenovirus (ADV), influenza A (FluA), influenza B (FluB) and respiratory syncytial virus (RSV) are common viruses that can lead to pediatric respiratory tract infections and further cause local epidemics, especially in schools, kindergartens and other places with relatively high population density. ADV can cause upper respiratory tract infections, conjunctivitis and severe pneumonia. Pneumonia caused by ADV infection accounts for 4–10 % of childhood viral pneumonia, and is one of the most serious types of childhood pneumonia [2]. Influenza viruses (IV) that infect humans are divided into three types: A, B and C. Among them, FluA often appears in epidemic form and can cause a worldwide influenza pandemic. FluB virus often causes local outbreaks. Type C influenza virus is rare. It often causes lower respiratory tract infections such as bronchitis, bronchiolitis, and pneumonia in children. The variation of influenza strains and the differences in epidemic strains in different regions have brought great challenges to influenza prevention [3]. RSV can infect the upper and lower respiratory tract at the same time. When only the upper respiratory tract is infected, the main symptoms are nasal congestion, runny nose and cough. When the virus invades the lower respiratory tract, it can cause bronchitis, bronchiolitis and pneumonia. In most studies, RSV was found to be the predominant viral cause of acute lower respiratory tract infections (ALRI) in childhood, being responsible for 27–96 % of hospitalized cases (mean 65 %) in which a virus was found [4]. The peak of respiratory diseases in children normally occurs in winter and spring seasons. Particularly, infections of RSV and influenza virus have seasonal variation with high incidence in winter and spring [5], whereas ADV can occur all the year round [6].

The outbreak of Corona Virus Disease 2019 (COVID-19) occurred in December 2019. Since January 23, 2020, a series of strict and active prevention and control measures had been proposed by Chinese government, such as isolation and wearing masks [7]. The Lancet reported that China had successfully contained infection of severe acute respiratory syndrome coronavirus 2 (SARS-CoV-2) and nearly stopped indigenous transmission by containment and suppression [8]. During the epidemic period, both the operation mechanism of the society and the behavior habits of individuals changed. Regarding the group of children, most of them were restricted to staying at home, and were asked to routinely wear masks and frequently wash hands.

Droplet transmission is the primary route to transmit respiratory tract infections. Isolating at home, reducing non-essential activities outside the home and wearing masks in public areas can greatly block the transmission routes of respiratory tract virus infections. The purpose of our study is to investigate the influence of isolation and the changed behavioral habits on respiratory tract infections in children through a retrospective study during the period of COVID-19 outbreak (January-April 2020). For comparison, the corresponding data in the same periods in 2018 and 2019 were utilized.

### Methods

Data on outpatient visits and respiratory infection visits, number of completed tests for respiratory viruses (RSV, ADV, FluA, FluB), and the tested positive cases were extracted from electronic healthcare records in the Children’s Hospital, Zhejiang University School of Medicine from January to April, 2020, compared with those in 2018 and 2019 during the same periods. The study was approved by the ethics committee of the Children’s Hospital of Zhejiang University School of Medicine (2020-IRB-016).

### Clinical variables

Clinical information including date of birth, gender, department of treatment, disease diagnosis and date of respiratory virus sampling was collected from all outpatients. Age was calculated by the difference between date of birth and date of sampling. In this study, respiratory tract infections included upper and lower respiratory tract infections. Upper respiratory tract infections included common cold, viral pharyngitis, laryngitis, herpangina, and tonsillitis. Lower respiratory infections included pneumonia, bronchitis, asthmatic bronchitis, and bronchiolitis [9].

### Tests of respiratory viruses

 Samples were obtained using the throat swab and were stored at 2 to 8 °C for up to 8 h until they were tested. Throat swabs should not be touched with saliva. Adenovirus was detected by using a colloidal gold method (diagnostic kit provided by KaiBiLi Company). RSV was tested by a diagnostic kit (colloidal gold method, KaiBiLi), which could also identify type A and type B, but was unable to distinguish the subtypes. Influenza A and B were detected using nucleoprotein antigen test kit (colloidal gold method, KaiBiLi).

### Statistical analysis

Categorical data was expressed in proportion. Differences of proportion in the same periods of 2018–2020 were compared using the Chi-square test. The age characteristic across different years was expressed as median (interquartile range, IQR) for non-parametrical distribution, and the statistical difference was compared by Wilcoxon rank sum test. All tests were two sided, and *P* < 0.05 was considered statistically significant. All statistical analyses were performed using R software (version 4.0.0).

## Results

### Comparing pediatric outpatient visits during the COVID-19 outbreak with those in the same periods in 2018 and 2019

**Fig. 1:**
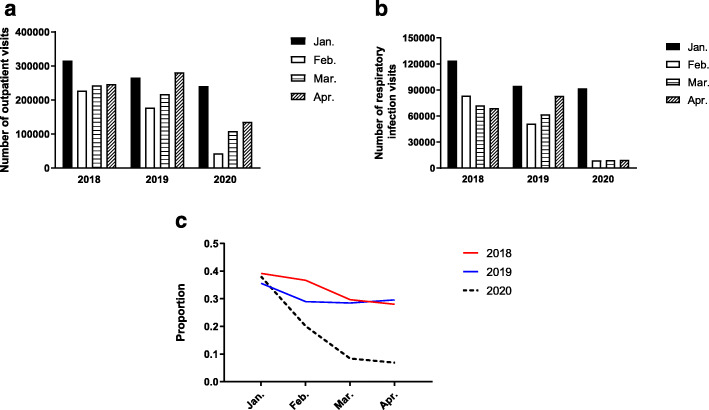
Distribution of outpatient visits and respiratory infections from January to April 2018-2020. **a**: Pediatric outpatient visits. **b**: The number of respiratory infection visits. **c**: The proportion of patients with respiratory infections in outpatient visits

From January to April, the numbers of outpatient visits in 2018 were 315,685, 227,398, 243,546, 247,039, and were 266,190, 177,377, 217,870, 281,457 in 2019, while in 2020 were only 241,251, 43,306, 108,989, 135,708, respectively. We found that outpatient visits in January, 2020 was comparable with those in 2018 and 2019, but decreased by 59.9% (288,003 vs. 717,983) and 57.4% (288,003 vs. 676,704), respectively during the period of February-April, 2020, as compared with the same periods in 2018 and 2019. In February of every year, outpatient visits declined significantly due to the Spring Festival, compared with other three months (Table 1; Fig. 1 a).

**Table 1:** The proportion of respiratory infections and outpatient visits from January to April in 2018-2020

Categories	2018	2019	2020	*p*
Age median (IQR) (years)	2 (0, 4)	2 (1, 4)	2 (1, 5)	< 0.001
Age ( n, %)				
≤ 3 (toddlers and infants)	247,538(71.0)	189,036 (64.8)	80,247 (67.1)	< 0.001
>3–6 (preschool children)	67,261 (19.3)	68,241 (23.4)	24,018 (20.1)	
≥ 6–18 ( school students)	33,963(9.7)	34,280 (11.8)	15,267 (12.8)	
Gender ( n, %)				
Male	197,058 (56.5)	162,112 (55.6)	65,245 (54.6)	< 0.001
Female	151,704 (43.5)	129,445 (44.4)	54,287 (45.4)	
Respiratory tract infection (n/N, %)				
Upper respiratory tract infection	176,672/348,762 (50.7)	139,481/291,557 (47.8)	6413/119,532 (5.4)	< 0.001
Lower respiratory tract infection	172,090/348,762 (49.3)	152,076/291,557 (52.2)	113,119/119,532 (94.6)	
Patients with respiratory tract infection /outpatient visits n/N (%)				
January	123,861/315,685(39.2)	94,862/266,190 (35.6)	91,911/241,251 (38.1)	< 0.001
February	83,448/227,398(36.7)	51,400/177,377 (29.0)	9093/43,306 (20.1)	< 0.001
March	72,383/243,546(29.7)	62,017/217,870 (28.5)	9137/108,989 (8.4)	< 0.001
April	69,070/247,039(28.0)	83,278/281,457 (29.6)	9391/135,708 (6.9)	< 0.001
Total	348,762/1,033,668(33.7)	291,557/942,894 (30.9)	119,532/529,254 (22.6)	< 0.001

*IQR* interquartile range; *p* < 0.05 was considered statistically different

Since January 23, 2020, the Chinese government had proposed immediate and strict epidemic prevention measures, such as isolation by staying at home and wearing masks. As compared with 2018 and 2019, pediatric outpatient visits in January 2020 was slightly lower than that in the previous periods, but was significantly reduced in February during the COVID-19 outbreak. As the epidemic continued after the Spring Festival, pediatric outpatient visits in March and April elevated slowly, but still lower than those in the same periods of 2018 and 2019 (Table 1; Fig. 1 a).

### Comparing number of pediatric respiratory infections during the COVID-19 outbreak with those in the same periods in 2018 and 2019

The total number of pediatric patients with respiratory infections from January to April 2020 was lower than that in 2018 and 2019, which decreased by 65.7 % (119,532 vs.348,762), 59.0 % (119,532 vs.291,557), respectively. From January to April, the number of patients with respiratory tract infections in 2020 was 91,911, 9093, 9137 and 9391 respectively, which decreased significantly since February. During the study periods, children and infants under 3 years were the majority of patients with respiratory tract infections, accounting for 71 %, 64.8 and 67.1 % in 2018, 2019 and 2020, respectively. The number of boys with respiratory infections was 197,058 (56.5 %), 162,112 (55.6 %) and 65,245 (54.6 %) in 2018, 2019 and 2020, respectively, and girls were 151,704 (43.5 %), 129,445 (44.4 %), and 54,287 (45.4 %). The number of upper and lower respiratory tract infections in 2020 was 6413 (5.4 %) and 113,119 (94.6 %), respectively, and the corresponding figure in 2018 was 176,672 (50.7 %) and 172,090 (49.3 %) and was 139,481 (47.8 %) and 152,076 (52.2 %) in 2019 (Table 1; Fig. 1b).

Affected by the epidemic, the proportion of patients with respiratory infections from January to April 2020 was lower than that in 2018 and 2019, especially in February, March and April (*P* < 0.001, Table 1; Fig. 1 c).

### Comparing positive cases of respiratory virus infections during the epidemic period with those in the same periods in 2018 and 2019

**Fig. 2:**
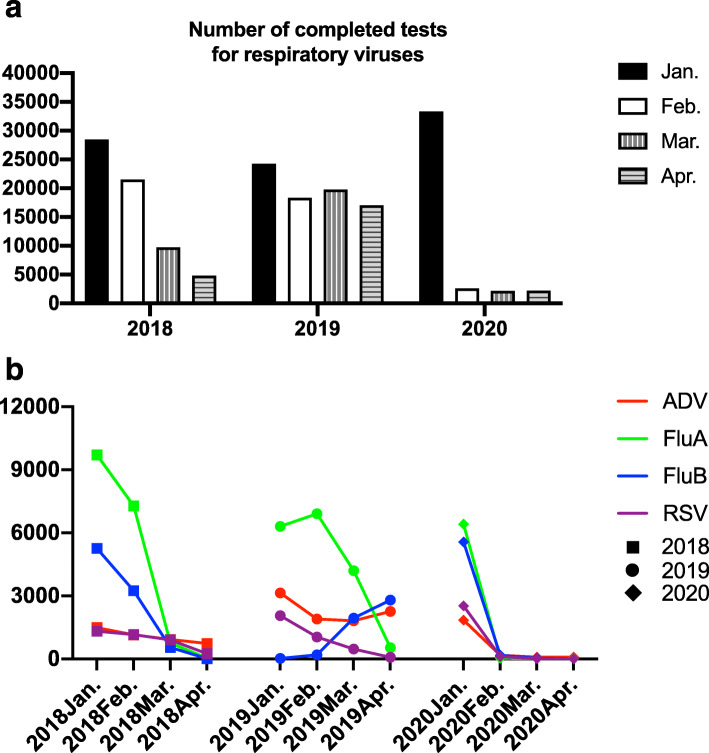
Distribution of positive cases of respiratory viruses from January to April 2018-2020. **a**: The number of completed tests for respiratory viruses. **b**: Distribution of positive cases of ADV, FluA, FluB, RSV from January to April in 2018, 2019 and 2020

During the COVID-19 outbreak, the number of completed tests for respiratory viruses declined significantly. From February to April, 2020, monthly number of completed tests for respiratory viruses was 2596, 2182 and 2227, respectively, which was lower than that in 2018 (21,490, 9731, 4816) and 2019 (18,317, 19,761, 17,025) (Fig. 2 a).

From January to April in 2020, ADV positive cases were 1853, 170, 95 and 94 respectively, which were lower than those in the same periods of 2018 (1484, 1151, 928, 737) and 2019 (3145, 1896, 1812, 2258) (Fig. 2b).

During the study period, FluA positive cases in 2020 were 6412, 39, 13 and 13, respectively. In February-April 2020, FluA positive cases were much lower than those in 2018 and 2019 (Fig. 2b).

During the study period, FluB positive cases in 2020 were 5565, 178, 34 and 8, respectively, which continued to decline. In February-April 2020, FluB positive cases were much lower than those in 2018 (Fig. 2b). At the beginning of 2020, FluB positive cases were relatively higher than those in 2019, but kept declining since the epidemic of COVID-19 in February (Fig. 2b).

From January to April in 2020, RSV positive cases were 2531, 114, 19 and 2, respectively. RSV positive cases from February to April in 2020 were lower than those in 2018 and 2019 (Fig. 2b).

## Discussion

In this study, we found that pediatric outpatient visits from January to April 2020 were significantly lower than those in the same periods of 2018 and 2019. The pediatric outpatient visits decreased in February and elevated slowly in March and April in 2020. Moreover, the number and proportion of pediatric respiratory tract infections significantly decreased during the outbreak. Consistently, a study from the Otolaryngology Department of Yale University Medical School reported that the planned visit completion rate was 12.9 % (649/5044) during the corresponding COVID-19 affected period, significantly lower than 62.0 % (3665/5913) in the same period of 2019, and 55.8 % of the visits completed in 2020 were conducted through telemedicine [10]. During the COVID-19 outbreak in Turkey, a study showed that outpatient visits in Department of Dermatology decreased significantly in a short time [11]. Two studies showed that during the COVID-19 pandemic in South America, due to the prevention and control of the epidemic, the number of respiratory infections decreased, resulting in a significant decrease in the number of Pediatric Intensive Care Unit (PICU) admissions [12, 13]. However, no studies were available for pediatric outpatient visits due to respiratory tract infections and related pathogens during the outbreak.

In our study, we also found that the number of tests for respiratory virus infections and the corresponding positive cases of ADV, FluA, FluB, and RSV decreased, especially in February 2020. ADV, FluA, FluB, and RSV are common viruses in children with respiratory tract infections. In Asia, the number of severe pneumonia caused by ADV infection has increased in recent years [14–16], with a high rate of fatality and sequelae for severe ADV infection [17, 18]. Influenza has a seasonal variation and is prevalent in winter and spring, and the virus strain is easy to mutate. Even with a vaccine, the epidemic occurs year after year, and commonly in schools and childcare institutions [19]. The epidemic of RSV has obvious climatic distribution characteristics with peak generally from December to February [20, 21], which is lacking of specific etiological treatment. Infants infected with RSV may have repeated coughing and wheezing, and eventually develop into childhood asthma [22]. SARS-CoV-2, ADV, FluA, FluB, and RSV are transmitted by contact, droplets and fomites. These viruses can cause respiratory diseases, which present as a wide range of illness from asymptomatic or mild through to severe disease and death. Isolation by staying at home and wearing masks are effective measures to prevent respiratory virus infections in children, because these measures could prevent entry of pathogens from the nose and mouth, reducing the respiratory tract infections in children (such as RSV, ADV, FluA and FluB). Previous studies have indicated that wearing masks, hand hygiene and keeping social distance might prevent viral infection [23, 24]. During the outbreak of COVID-19, a study showed that a COVID-19 patient from Chongqing, China did not wear a mask in one vehicle and transmitted to 5 persons, and no one was infected when he wore a mask in the second vehicle later [25]. Current evidence suggests that SARS-CoV-2 is mainly spread by respiratory droplets, which can enter body through the eyes, nose and mouth, or contacting with contaminated surfaces. A systematic review and meta-analysis published in the Lancet supported that physical distancing, face masks, and eye protection played important roles in preventing person-to-person transmission of COVID-19 [26]. A study found that the measures taken by the Chinese government to control SARS-CoV-2 also controlled the spread of influenza virus, as COVID-19 and influenza virus were infectious respiratory diseases with the same route and means of transmission [27]. Another study found after the implementation of public health measures for COVID-19, the incidence of influenza in Singapore declined significantly compared with that from 3 preceding years, and suggested that the measures taken for COVID-19 were effective in reducing transmission of other viral respiratory diseases [28].

From January to February 2020, there was a trend of rapid increase of disease onset in the early stage of the epidemic. The government implemented various measures to prevent the spread of the epidemic. Public gatherings were banned and significant events canceled. People were not allowed to enter in traffic ports, communities, hospitals or other public places without wearing a mask. Most public places were equipped with an alcohol-based hand rub to remind for hand hygiene. The workplaces were closed. The school class start dates were delayed, and primary and secondary school students took online courses at home. In terms of personal protection, adults and children should stay home, reduce unnecessary outings, wash hands frequently, wear a mask in public places, and keep a physical distance of at least 1 m from others to reduce risk of infection. From March to April, the epidemic in China was basically under control. Wearing a mask was still the most important preventive measure in closed or crowded spaces, such as elevators, transport, hospitals and schools, etc. The schools gradually resumed, and primary and middle school students were registered for temperature measurements and health status registrations every day. Once a student had a fever or respiratory symptoms, he must go to the fever clinic to be tested negative for SARS-CoV-2, isolate at home for 48 h before returning to school. Students who came from high-risk areas must be conducted home or centralized health surveillance for 14 days. The above measures were also applicable to the prevention of influenza and other respiratory diseases [29, 30]. In our study, we found that the proportion of pediatric patients with respiratory infections among total outpatients, the number of completed tests for respiratory viruses and the corresponding positive cases of ADV, FluA, FluB, and RSV did not elevate in March and April compared with the same periods in 2018 and 2019. The rate of respiratory tract infections was still lower in the “sequelae of isolation” or “post-isolation era”. This phenomenon could be explained by several reasons as follows: (1) Parents’ awareness of prevention for respiratory tract infections was enhanced and these preventive measures were maintained such as wearing masks, and keeping social distance; (2) Some patients were likely in the incubation period; (3) The incidence of respiratory infections was lower in warm seasons [31].

Respiratory tract infection in children has a characteristic of seasonal variation, with a high incidence in winter and spring. Outpatient service will be full in every winter and spring regardless of the major specialized children’s hospitals, pediatrics of general hospitals, or maternal and child health care hospitals. Patients are difficult to register and purchase drugs due to lack of pediatricians. In 2020, affected by the epidemic, the overall number of outpatients and the number and proportion of pediatric respiratory tract infections in children’s hospital decreased significantly. Personal protective measures such as isolation by staying at home, wearing masks and washing hands frequently have played a role in reducing cross infection and risk of respiratory tract infections to a great extent. It is concluded that the public should pay attention to public health and advocate prevention combined with treatment, rather than focusing on treatment but ignoring prevention [32].

## Conclusions

Our study illustrated, for the first time through the analysis of large samples, that during the COVID-19 epidemic, blocking transmission routes (including wearing masks, isolation by staying at home and frequent hand washing) might partly explain the reduction of pediatric outpatient visits for respiratory tract infections and the related respiratory diseases caused by common respiratory viruses, including ADV, FluA, FluB, and RSV. This study confirms the importance of transmission route blocking that can prevent pediatric respiratory tract infections. As the COVID-19 epidemic is still ongoing, we recommend reducing aggregated activities, wearing masks in public places, shutting down the school in serious conditions, and performing online education through internet-based courses. During the epidemic period of respiratory viruses, we recommend that the children should reduce gathering activities, wear masks, wash hands frequently, and actively protect children’s health and safety from SARS-CoV-2, influenza, ADV, or RSV virus infection.

## Data Availability

The data of the current study are available from the corresponding author on reasonable request.

## Abbreviations

ADV: Adenovirus
FluA: Influenza A
FluB: Influenza B
RSV: Respiratory syncytial virus
IV: Influenza viruses
ALRI: Acute lower respiratory tract infections
COVID-19: Corona Virus Disease 2019
SARS-CoV-2: Severe acute respiratory syndrome coronavirus 2
PICU: Pediatric Intensive Care Unit
